# Comorbility symptoms in AHDH adult patients

**DOI:** 10.1192/j.eurpsy.2022.1184

**Published:** 2022-09-01

**Authors:** P. Marqués Cabezas, A.I. Segura Rodríguez, P. García Barriuso, L. Gallardo Borge, M.J. Mateos Sexmero, J.A. Blanco, M. Queipo De Llano De La Viuda, M. Perez Carranza, A. Aparicio Parras, J. Espina Barrio, A. Rodriguez Campos

**Affiliations:** Hospital Clínico Universitario, Psychiatry, Valladolid, Spain

**Keywords:** ADULT PATIENTS, COMORBILITY, adhd, Psychosis

## Abstract

**Introduction:**

Adults may continue suffering AHDH symptoms after this condition is recognized and typified in children. Different works provide evidence that adults have an even more complicated variety of psychiatric disorders than children, as an increased risk of problems stemming from substance abuse, depression, anxiety, increased risk of traffic accidents, and also sexual transmission diseases.

**Objectives:**

There was known that adults could continue suffering symptoms derived from his infantile ADHD. We wonder if the majority of the young males derived to our consultation present compatible symptoms with adult ADHD. This condition promotes the onset of substance use and may lead to latent psychosis onset.

**Methods:**

We analyzed 39 patients derived by suspicion of psychiatric pathology, aged between 17 and 35. They stem to clinical psychology for study of features of personality (Million Questionnaire). Another questionnaire was used also autoapplied for sifted of the ADHD in adults (ASRS_V1:1). According to the criteria DSM-IV TR, the patient had moderate symptoms of ADHD if it was fulfilling 6 or more diagnostic criteria according to their answers in the screening questionnaire.

**Results:**

The results supported the existence of impulsivity, aggression, irritability, problems with compliance and substance abuse.

**Conclusions:**

ADHD is not only a problem of distractibility or worry, but a deeper and extensive alteration caused by the deterioration of a set of cerebral activities. An early treatment in the childhood could prevent devastating consequences for their development, since they include the majority of the functional areas of the patient and it impedes their later social and labor adjustment.

**Disclosure:**

No significant relationships.

